# Epidural Steroid Injection Prior to Spinal Surgery: A Step-Wise and Wise Approach

**DOI:** 10.7759/cureus.45125

**Published:** 2023-09-12

**Authors:** Michael Kozak, David R Hallan, Elias Rizk

**Affiliations:** 1 Neurosurgery, Penn State College of Medicine, Hershey, USA; 2 Neurological Surgery, Penn State Health Milton S. Hershey Medical Center, Hershey, USA

**Keywords:** dural tear, spinal abscess, spinal surgery, epidural steroid injection, esi

## Abstract

Background: An epidural steroid injection (ESI) is used to treat a number of morbid central nervous system pathologies and is considered a reasonably safe procedure. This study aimed to determine the relative infection risk after spinal surgery by comparing outcomes in spinal surgery patients who received an ESI shortly prior to the surgery against those who did not receive an ESI shortly prior to the surgery.

Methods: The present study is a retrospective cohort study using a multi-institutional healthcare database, TriNetX, to collect data on patients who received spinal surgery with and without having had ESIs six months before surgery. Two cohorts were generated: Cohort 1 included patients who had received an ESI in the six months prior to spinal surgery, and cohort 2 included patients who did not have an ESI in the six months prior to spinal surgery. The patients in cohort 2 had propensity scores matched 1:1 to those in cohort 1 using common baseline demographics, comorbidities and spinal procedure indications. The spinal procedures and surgeries considered for the analysis included open procedures for any purpose, including exploration, decompression, resection, revision or biopsy. Multiple outcomes were compared across these two cohorts in the three months following the spinal procedure/surgery, including the occurrence of death, surgical site infection, epidural and/or spinal abscess, and dural tear.

Results: An ESI in the six months prior to spinal surgery was associated with a significant decrease in the likelihood epidural/spinal abscess in the three months after surgery. There was no change in mortality, wound infection or identification of dural tear in the three months after spinal surgery for those who received an ESI six months before spinal surgery.

Conclusion: This data suggests that epidural steroid injections' anti-inflammatory effects provide benefits before surgery beyond symptomatic relief. Given that the degeneration of spinal pathologies is typically advanced rather than corrected by the body's inflammatory response, it is likely that preventing hyperactivation of the body's immune system in the months preceding surgical intervention, a traumatic insult, is protective compared to no intervention and, importantly, without major adverse effects.

## Introduction

Epidural steroid injections (ESIs) have been in use for more than 50 years to treat a number of morbid central nervous system (CNS) pathologies, including disc herniation, spinal arthritis, discogenic pain and others. Chronic low back and neck pain are two of the most morbid conditions of modern times [1]. Epidural steroid injections to the spine deliver corticosteroids directly above the dura, just deep to the ligamentum flavum, to facilitate localized anti-inflammatory effects and reduce the compression of nerves and the spinal cord to relieve symptoms temporarily. Epidural steroid injections may be classified by location (cervical, thoracic, or lumbar) and the needle's path (interlaminar, transforaminal, or caudal) [2]. An ESI is considered a temporizing measure for pain; however, there is not good evidence for its efficacy [3-4]. Additionally, it is not a permanent solution [5]. For most patients without traditional 'red flag' symptoms such as weakness, bowel/bladder incontinence, or saddle anesthesia, the traditional management of patients presenting with back pain begins with physical therapy and non-steroidal anti-inflammatory drugs (NSAIDs), and if these fail, often an ESI is trialed prior to the surgery. While there is no consensus as to the timing, the frequency or duration of ESI treatment for most spinal pathologies, more than half of surgeons report offering an ESI when other conservative methods fail, prior to proposing surgical intervention [6].

An ESI is considered a reasonably safe procedure. However, it is associated with several complications, such as hematoma, allergic reactions, and infection [7]. Infections can worsen a patient's condition from their pre-procedure baseline. Likewise, infections after spinal surgery have a considerable impact on recovery. The reported incidence of surgical site infection after spinal surgery ranges substantially depending on the indication and procedure (0.5%-20%). In contrast, less invasive procedures tend to have lower rates of post-procedural infection [8].

Spinal epidural injection is the precedent intervention to a considerable number of sub-acute spinal surgeries; for example, more than 45% cases of lumbar spinal stenosis or disc herniation received ESIs in the 12 months prior to surgery [9]. For this reason, there is considerable interest in the effect of an ESI on post-surgical outcomes for those receiving ESIs before surgery. The rationale is that the epidural space was only recently invaded by a delivery needle delivering anti-inflammatory steroids, thus creating functional local immunosuppression with potential tissue damage.

This study aims to leverage a large, multi-institutional healthcare database of patient information to determine the relative infection risk of spinal surgery when an ESI was given shortly prior to surgery by comparing outcomes in spinal surgery patients who received an ESI shortly prior to surgery versus those who did not receive an ESI shortly prior to surgery.

## Materials and methods

The present study is a retrospective cohort study using a multi-institutional healthcare database, TriNetX, to collect data from up to 102 different healthcare organizations (HCOs) on patients who received spinal surgery with and without having had epidural steroid injections six months before surgery. The TriNetX United States (US) Collaborative Network, containing information from 86,401,400 de-identified patients collected over 20 years, was accessed on May 29, 2023, to stratify patients into two cohorts. Cohort 1 included patients who had received an ESI in the six months prior to spinal surgery, and Cohort 2 included patients who did not have an ESI in the six months prior to spinal surgery. A period of six months was chosen because other studies have indicated that six months is the longest expected time for improved functional status after an ESI and because the Centers for Medicare & Medicaid Services supports no more than four ESI injections over any rolling 12-month period [10-11]. The patients in cohort 2 also had propensity scores matched 1:1 to those in cohort 1, using common baseline demographics and comorbidities such as age, gender, ethnicity, race, medication-related immunodeficiency, as well as medical histories including cardiovascular disease, alcohol use, tobacco use, diabetes, atrial fibrillation, hypertensive disorders and traumatic central nervous system injuries. Additionally, indications for an epidural steroid injection and/or spinal surgery were propensity matched for the most common indications including radiculopathy, osteoporosis, degenerative disc disease, primary neoplasm of the spinal cord and meninges, metastases to spinal cord and meninges, discitis, vertebral fractures, spinal stenosis, cauda equina and conus medullaris among other spinal pathologies. The index date was set as the date of spinal surgery. The TriNetX database is federated. Thus, an institutional review board approval for this study has been waived.

The spinal procedures and surgeries considered for the analysis included open, endoscopic and percutaneous procedures for any purpose, including exploration, decompression, resection, revision or biopsy. They included the following procedures at all levels along the spinal cord: discectomy, laminectomy, facetectomy, foraminotomy, decompression, release, arthrodesis, excision, biopsy, and resection.

Multiple outcomes were compared across these two cohorts in the three months following the spinal procedure/surgery, including the occurrence of death, surgical site infection, epidural and/or spinal abscess, and dural tear.

All statistical analyses were performed using the TriNetX online platform. Descriptive measures, such as means with standard deviations and proportions, were used to describe patient characteristics. For each outcome, the risk ratio, relative risk, and odds ratio were calculated to estimate the effects of a recent ESI on the outcomes. An a priori-defined two-sided alpha value of <0.05 was used for statistical significance.

## Results

Among all qualifying patients in the database, 23,549 patients (cohort 1) underwent a spinal surgery or procedure six months after receiving a spinal epidural injection. In addition, 389,916 patients in the database underwent a spinal procedure without receiving a spinal epidural injection in the preceding six months. Mean age, gender ratio, ethnicity, and comorbid conditions were substantially dissimilar in both groups before propensity matching (Table 1).

**Table 1:** Baseline patient characteristics of cohorts before propensity score matching ESI, epidural steroid injection; ICD-10, International Classification of Diseases, 10th Revision Cohort 1 = ESI in the six months leading up to surgery Cohort 2 = no ESI in the six months leading up to surgery Code is the ICD-10 code from patient charts for diagnoses, procedures and medications.

Baseline characteristics before propensity matching							
Demographics							
Cohort	Code	Characteristic	Mean ± SD	Patients	% of cohort	p-value	Std. diff.
1	AI	Age at index (years)	57.8 ± 15.2	23,549	100	<0.001	0.045
2			57.1 ± 16.9	389,916	100		
1	2106-3	White	-	18,689	79.4	<0.001	0.265
2				264,327	67.8		
1	F	Female	-	11,481	48.8	<0.001	0.048
2				180,789	46.4		
1	2054-5	Black or African American	-	1,400	5.9	<0.001	0.104
2				33,684	8.6		
1	2028-9	Asian	-	359	1.5	<0.001	0.072
2				9,886	2.5		
1	M	Male	-	12,068	51.2	<0.001	0.046
2				208,779	53.5		
1	2131-1	Unknown race	-	2,991	12.7	<0.001	0.072
2				78,862	20.2		
Diagnoses							
Cohort	Code	Characteristic		Patients	% of cohort	p-value	Std. diff.
1	I10-I16	Hypertensive diseases		10,561	44.8	<0.001	0.120
2				151,775	38.9		
1	E78	Disorders of lipoprotein metabolism and other lipidemias		7,899	33.5	<0.001	0.178
2				99,225	25.4		
1	E08-E13	Diabetes mellitus		3,675	15.6	<0.001	0.010
2				59,509	15.3		
1	E65-E68	Overweight, obesity and other hyperalimentation		4,396	18.7	<0.001	0.115
2				56,147	14.4		
1	N17-N19	Acute kidney failure and chronic kidney disease		1,435	6.1	0.102	0.011
2				24,803	6.4		
1	Z87.891	Personal history of nicotine dependence		2,679	11.4	<0.001	0.040
2				39,551	10.1		
1	F17	Nicotine dependence		2,987	12.7	<0.001	0.038
2				44,681	11.5		
1	F10.1	Alcohol abuse		280	1.2	<0.001	0.028
2				5,876	1.5		
1	F10.2	Alcohol dependence		171	0.7	0.001	0.024
2				3,681	0.9		
1	J40-J47	Chronic lower respiratory diseases		3,186	13.5	<0.001	0.057
2				45,386	11.6		
1	I48	Atrial fibrillation and flutter		906	3.8	0.004	0.020
2				16,537	4.2		
1	I50	Heart failure		574	2.4	<0.001	0.037
2				11,838	3.0		
1	I20-I25	Ischemic heart diseases		2,433	10.3	<0.001	0.028
2				36,985	9.5		
1	I73	Other peripheral vascular diseases		645	2.7	<0.001	0.040
2				8,281	2.1		
1	R40	Somnolence, stupor and coma		172	0.7	<0.001	0.081
2				6,244	1.6		
1	R53	Malaise and fatigue		1,692	7.2	0.743	0.002
2				27,795	7.1		
1	R13	Aphagia and dysphagia		380	1.6	<0.001	0.092
2				11,682	3.0		
1	S06.6	Traumatic subarachnoid hemorrhage		10	0.0	<0.001	0.074
2				1,500	0.4		
1	S06.3	Focal traumatic brain injury		10	0.0	<0.001	0.050
2				878	0.2		
1	S06.4	Epidural hemorrhage		12	0.1	<0.001	0.084
2				1,880	0.5		
1	S06.1	Traumatic cerebral edema		10	0.0	0.173	0.010
2				256	0.1		
1	R51	Headache		911	3.9	<0.001	0.048
2				11,692	3.0		
1	D84.9	Immunodeficiency, unspecified		71	0.3	0.004	0.018
2				829	0.2		
1	Z79.899	Other long-term (current) drug therapy		3,368	14.3	<0.001	0.124
2				39,895	10.2		
1	M54.5	Low back pain		12,658	53.8	<0.001	0.701
2				84,531	21.7		
1	M54.1	Radiculopathy		17,447	74.1	<0.001	0.983
2				117,104	30.0		
1	M81.0	Age-related osteoporosis without current pathological fracture		1,078	4.6	<0.001	0.083
2				11,658	3.0		
1	M51.36	Other intervertebral disc degeneration, lumbar region		9,376	39.8	<0.001	0.563
2				60,633	15.6		
1	M47.816	Spondylosis without myelopathy or radiculopathy, lumbar region		4,434	18.8	<0.001	0.310
2				32,449	8.3		
1	M51.16	Intervertebral disc disorders with radiculopathy, lumbar region		5,202	22.1	<0.001	0.413
2				30,018	7.7		
1	C61	Malignant neoplasm of prostate		312	1.3	<0.001	0.025
2				4,125	1.1		
1	C79.31	Secondary malignant neoplasm of brain		33	0.1	<0.001	0.050
2				1,549	0.4		
1	C79.49	Secondary malignant neoplasm of other parts of nervous system		32	0.1	<0.001	0.081
2				2,500	0.6		
1	C79.32	Secondary malignant neoplasm of cerebral meninges		18	0.1	0.433	0.005
2				360	0.1		
1	M47	Spondylosis		11,832	50.2	<0.001	0.347
2				130,142	33.4		
1	M48.00	Spinal stenosis, site unspecified		1,571	6.7	<0.001	0.128
2				14,880	3.8		
1	M47.819	Spondylosis without myelopathy or radiculopathy, site unspecified		522	2.2	<0.001	0.115
2				3,172	0.8		
1	M46.4	Discitis, unspecified		594	2.5	0.001	0.022
2				8,517	2.2		
1	M46.47	Discitis, unspecified, lumbosacral region		505	2.1	<0.001	0.102
2				3,485	0.9		
1	M46.40	Discitis, unspecified, site unspecified		56	0.2	<0.001	0.050
2				2,136	0.5		
1	M46.46	Discitis, unspecified, lumbar region		27	0.1	<0.001	0.068
2				1,904	0.5		
1	M46.45	Discitis, unspecified, thoracolumbar region		21	0.1	<0.001	0.030
2				796	0.2		
1	D49.2	Neoplasm of unspecified behavior of bone, soft tissue, and skin		98	0.4	<0.001	0.100
2				5,258	1.3		
1	C72.0	Malignant neoplasm of spinal cord		10	0.0	<0.001	0.074
2				1,501	0.4		
1	D21	Other benign neoplasms of connective and other soft tissue		49	0.2	0.789	0.002
2				780	0.2		
1	C49	Malignant neoplasm of other connective and soft tissue		32	0.1	0.008	0.020
2				851	0.2		
1	S32.0	Fracture of lumbar vertebra		410	1.7	<0.001	0.076
2				11,206	2.9		
1	S12	Fracture of cervical vertebra and other parts of neck		205	0.9	<0.001	0.179
2				13,528	3.5		
1	S32	Fracture of lumbar spine and pelvis		503	2.1	<0.001	0.077
2				13,245	3.4		
1	S12.9XXA	Fracture of neck, unspecified, initial encounter		60	0.3	<0.001	0.125
2				5,370	1.4		
1	S82.11	Fracture of tibial spine		10	0.0	0.117	0.009
2				99	0.0		
1	G83.4	Cauda equina syndrome		254	1.1	<0.001	0.060
2				7,001	1.8		
1	M48.0	Spinal stenosis		16,881	71.7	<0.001	0.344
2				215,932	55.4		
1	G95.81	Conus medullaris syndrome		10	0.0	0.015	0.019
2				354	0.1		
1	G95.89	Other specified diseases of spinal cord		258	1.1	<0.001	0.121
2				10,712	2.7		
Procedures							
Cohort	Code	Characteristic		Patients	% of cohort	p-value	Std. diff.
1	31500	Intubation, endotracheal, emergency procedure		11	0.0	<0.001	0.081
2				1,759	0.5		
Medications							
Cohort	Code			Patients	% of cohort	p-value	Std. diff.
1	11289	Warfarin		233	1.0	0.374	0.006
2				3,634	0.9		
1	1364430	Apixaban		185	0.8	0.256	0.008
2				3,336	0.9		
1	1114195	Rivaroxaban		120	0.5	0.868	0.001
2				2,018	0.5		
1	1191	Aspirin		3,860	16.4	<0.001	0.138
2				45,245	11.6		
1	32968	Clopidogrel		290	1.2	0.002	0.022
2				5,795	1.5		
1	3521	Dipyridamole		19	0.1	0.408	0.006
2				382	0.1		
1	613391	Prasugrel		13	0.1	0.952	<0.001
2				219	0.1		

After propensity-matched data analysis, 23,539 patients remained in both cohorts (Table 2). Therefore, the reported analyses were performed after propensity matching. The results are presented in Table 3.

**Table 2:** Patient demographics, diagnoses, procedures and medications for cohorts after propensity matching ESI, epidural steroid injection; ICD-10, International Classification of Diseases, 10th Revision Cohort 1 = ESI in the six months leading up to surgery Cohort 2 = no ESI in the six months leading up to surgery Code is the ICD-10 code from patient charts for diagnoses, procedures and medications.

Characteristics after propensity matching							
Demographics							
Cohort	Code	Characteristic	Mean ± SD	Patients	% of cohort	p-value	Std. diff.
1	AI	Age at index (years)	57.8 ± 15.2	23,539	100	0.573	0.005
2			57.7 ± 15.0	23,539	100		
1	2106-3	White	-	18,679	79.4	0.057	0.004
2				18,845	80.1		
1	F	Female	-	11,471	48.7	0.678	0.006
2				11,426	48.5		
1	2054-5	Black or African American	-	1,400	5.9	0.531	0.006
2				1,368	5.8		
1	2028-9	Asian	-	359	1.5	0.319	0.009
2				333	1.4		
1	M	Male	-	12,068	51.3	0.782	0.003
2				12,098	51.4		
1	2131-1	Unknown race	-	2,991	12.7	0.125	0.014
2				2,881	12.2		
Diagnoses							
Cohort	Code	Characteristic		Patients	% of cohort	p-value	Std. diff.
1	I10-I16	Hypertensive diseases		10,559	44.9	0.001	0.032
2				10,186	43.3		
1	E78	Disorders of lipoprotein metabolism and other lipidemias		7,894	33.5	<0.001	0.036
2				7,496	31.8		
1	E08-E13	Diabetes mellitus		3,675	15.6	0.008	0.024
2				3,469	14.7		
1	E65-E68	Overweight, obesity and other hyperalimentation		4,395	18.7	0.001	0.030
2				4,127	17.5		
1	N17-N19	Acute kidney failure and chronic kidney disease		1,433	6.1	0.013	0.023
2				1,307	5.6		
1	Z87.891	Personal history of nicotine dependence		2,679	11.4	0.003	0.028
2				2,475	10.5		
1	F17	Nicotine dependence		2,987	12.7	0.412	0.008
2				2,928	12.4		
1	F10.1	Alcohol abuse		280	1.2	0.277	0.010
2				255	1.1		
1	F10.2	Alcohol dependence		171	0.7	0.870	0.002
2				168	0.7		
1	J40-J47	Chronic lower respiratory diseases		3,185	13.5	0.004	0.026
2				2,977	12.6		
1	I48	Atrial fibrillation and flutter		906	3.8	0.181	0.012
2				851	3.6		
1	I50	Heart failure		574	2.4	0.026	0.020
2				502	2.1		
1	I20-I25	Ischemic heart diseases		2,432	10.3	0.017	0.022
2				2,277	9.7		
1	I73	Other peripheral vascular diseases		645	2.7	0.569	0.005
2				625	2.7		
1	R40	Somnolence, stupor and coma		172	0.7	0.089	0.016
2				142	0.6		
1	R53	Malaise and fatigue		1,692	7.2	0.001	0.030
2				1,516	6.4		
1	R13	Aphagia and dysphagia		380	1.6	0.030	0.020
2				323	1.4		
1	S06.6	Traumatic subarachnoid hemorrhage		10	0.0	1	<0.001
2				10	0.0		
1	S06.3	Focal traumatic brain injury		10	0.0	1	<0.001
2				10	0.0		
1	S06.4	Epidural hemorrhage		12	0.1	0.670	0.004
2				10	0.0		
1	S06.1	Traumatic cerebral edema		10	0.0	1	<0.001
2				10	0.0		
1	R51	Headache		911	3.9	0.208	0.012
2				859	3.6		
1	D84.9	Immunodeficiency, unspecified		70	0.3	0.015	0.022
2				44	0.2		
1	Z79.899	Other long-term (current) drug therapy		3,367	14.3	0.001	0.030
2				3,126	13.3		
1	M54.5	Low back pain		12,648	53.7	0.305	0.009
2				12,759	54.2		
1	M54.1	Radiculopathy		17,437	74.1	0.220	0.011
2				17,320	73.6		
1	M81.0	Age-related osteoporosis without current pathological fracture		1,075	4.6	0.773	0.003
2				1,062	4.5		
1	M51.36	Other intervertebral disc degeneration, lumbar region		9,366	39.8	0.821	0.002
2				9,342	39.7		
1	M47.816	Spondylosis without myelopathy or radiculopathy, lumbar region		4,427	18.8	0.169	0.013
2				4,311	18.3		
1	M51.16	Intervertebral disc disorders with radiculopathy, lumbar region		5,192	22.1	0.338	0.009
2				5,106	21.7		
1	C61	Malignant neoplasm of prostate		312	1.3	0.285	0.010
2				286	1.2		
1	C79.31	Secondary malignant neoplasm of brain		33	0.1	0.102	0.015
2				21	0.1		
1	C79.49	Secondary malignant neoplasm of other parts of nervous system		32	0.1	0.605	0.005
2				28	0.1		
1	C79.32	Secondary malignant neoplasm of cerebral meninges		18	0.1	0.130	0.014
2				10	0.0		
1	M47	Spondylosis		11,822	50.2	<0.001	0.041
2				11,342	48.2		
1	M48.00	Spinal stenosis, site unspecified		1,568	6.7	0.218	0.011
2				1,502	6.4		
1	M47.819	Spondylosis without myelopathy or radiculopathy, site unspecified		519	2.2	0.613	0.005
2				503	2.1		
1	M46.4	Discitis, unspecified		592	2.5	0.573	0.005
2				573	2.4		
1	M46.47	Discitis, unspecified, lumbosacral region		503	2.1	0.653	0.004
2				489	2.1		
1	M46.40	Discitis, unspecified, site unspecified		56	0.2	0.465	0.007
2				64	0.3		
1	M46.46	Discitis, unspecified, lumbar region		27	0.1	0.599	0.005
2				31	0.1		
1	M46.45	Discitis, unspecified, thoracolumbar region		21	0.1	0.117	0.014
2				12	0.1		
1	D49.2	Neoplasm of unspecified behavior of bone, soft tissue, and skin		98	0.4	0.610	0.005
2				91	0.4		
1	C72.0	Malignant neoplasm of spinal cord		10	0.0	0.670	0.004
2				12	0.1		
1	D21	Other benign neoplasms of connective and other soft tissue		49	0.2	0.692	0.004
2				53	0.2		
1	C49	Malignant neoplasm of other connective and soft tissue		32	0.1	0.354	0.009
2				25	0.1		
1	S32.0	Fracture of lumbar vertebra		410	1.7	0.026	0.021
2				349	1.5		
1	S12	Fracture of cervical vertebra and other parts of neck		205	0.9	0.016	0.022
2				159	0.7		
1	S32	Fracture of lumbar spine and pelvis		503	2.1	0.062	0.017
2				446	1.9		
1	S12.9XXA	Fracture of neck, unspecified, initial encounter		60	0.3	0.010	0.024
2				35	0.1		
1	S82.11	Fracture of tibial spine		10	0.0	1	<0.001
2				10	0.0		
1	G83.4	Cauda equina syndrome		254	1.1	0.078	0.016
2				216	0.9		
1	M48.0	Spinal stenosis		16,872	71.7	0.002	0.028
2				16,568	70.4		
1	G95.81	Conus medullaris syndrome		10	0.0	1	<0.001
2				10	0.0		
1	G95.89	Other specified diseases of spinal cord		258	1.1	0.059	0.017
2				217	0.9		
Procedures							
Cohort	Code	Characteristic		Patients	% of cohort	p-value	Std. diff.
1	31500	Intubation, endotracheal, emergency procedure		11	0.0	0.827	0.002
2				10	0.0		
Medications							
Cohort	Code			Patients	% of cohort	p-value	Std. diff.
1	11289	Warfarin		233	1.0	0.046	0.018
2				192	0.8		
1	1364430	Apixaban		185	0.8	0.958	<0.001
2				186	0.8		
1	1114195	Rivaroxaban		120	0.5	0.426	0.007
2				108	0.5		
1	1191	Aspirin		3,852	16.4	0.058	0.017
2				3,701	15.7		
1	32968	Clopidogrel		290	1.2	0.121	0.014
2				254	1.1		
1	3521	Dipyridamole		19	0.1	0.639	0.004
2				22	0.1		
1	613391	Prasugrel		13	0.1	0.841	0.002
2				12	0.1		

**Table 3:** Outcomes for cohorts after propensity matching, comparing rates of death, wound infection, dural tear and epidural and/or spinal abscess

Outcome	Cohort	No. of patients	Patients with the outcome	Percentage of cohort	Risk difference	95% CI	p-value
Deceased	1	23,539	126	0.535	0.000	-0.001 to 0.002	0.563
	2	23,539	217	0.497			
Wound infection	1	22,276	350	1.571	-0.001	-0.003 to 0.002	0.609
	2	22,428	366	1.632			
Dural tear	1	22,945	92	0.401	0.001	0.0 to 0.002	0.103
	2	22,880	71	0.310			
Epidural and/or spinal abscess	1	23,539	115	0.489	-0.003	-0.005 to -0.002	0.000
	2	23,539	189	0.803			

The risk of mortality in the six months after spinal surgery was not statistically significantly different between the cohorts, with 126 of 23,539 (0.535%) patients in the ESI before the spinal procedure group (cohort 1) and 217 of 23,539 (0.497%) patients in the non-ESI cohort dying in the six months after spinal surgery.

The risk of experiencing a wound infection in the six months after surgery was not statistically significantly different between the cohorts; 350 of 22,276 (1.571%) patients in the ESI cohort (cohort 1) and 366 of 22,428 (1.632%) of those who did not receive an ESI prior to surgery (cohort 2) experienced a wound infection in the six months after spinal surgery. The risk difference of having a wound infection was -0.1% (-0.3% to 0.2%, 95% CI; p = 0.609) between the cohorts.

The risk of diagnosing a dural tear in the six months after surgery was not statistically significantly different between the cohorts; 92 of 22,945 (0.401%) patients in the ESI cohort (cohort 1) and 71 of 22,880 (0.310%) of those who did not receive an ESI prior to surgery (cohort 2) were diagnosed with a dural tear in the six months after spinal surgery. The risk difference of a dural tear was 0.1% (0.0% to 0.2%, 95% CI; p = 0.103) between the cohorts.

The risk of experiencing an epidural and/or spinal abscess in the six months after surgery was statistically significantly different between the cohorts; 115 of 23,539 (0.489%) patients in the ESI cohort (cohort 1) and 189 of 23,539 (0.803%) of those who did not receive ESI prior to surgery (cohort 2) experienced an epidural and/or spinal abscess in the six months after spinal surgery. The risk difference of having an epidural or spinal abscess was -0.3% (-0.5% to -0.2%, 95% CI; p = 0.000) between the cohorts.

## Discussion

Several studies have looked at specific surgical outcomes in patients previously treated with ESIs. These studies have generated conflicting evidence. For instance, two studies, one a retrospective Medicare analysis and the other a large insurance database retrospective cohort analysis demonstrated that an ESI in the preceding months of surgery was associated with a statistically significant increased risk of postoperative infection for single-level primary lumbar decompression without fusion. However, the risk of infection was still less than 2% in the ESI group [12-13]. However, an analysis of patients in the Military Health System undergoing single-level lumbar decompression found no difference in postoperative infection rates between those who had recently had an ESI and those who had not [14]. Another national insurance database retrospective study found that an ESI in the three months before lumbar fusion surgery significantly increased the risk of postoperative infection [15]. 

Many previous studies have been specific about the procedural site and indication [12-15]. To interrogate the relationship more robustly between ESIs and post-surgical outcomes, the present study expanded the scope of post-surgical outcomes for patients receiving an ESI and broadened the surgeries included. The present findings suggest no mortality benefit nor was there a decreased likelihood of a dural tear or wound infection. The present study did find a statistically significant decrease in the likelihood epidural/spinal abscess in the three months after spinal surgery for patients who received ESIs in the six months prior to surgery. This data suggests that epidural steroid injections' anti-inflammatory effects provide benefits, and likely no harm, before surgery beyond symptomatic relief. Given that degeneration of anatomic spinal pathologies is potentially advanced rather than corrected by the body's inflammatory response [16], it is likely that preventing hyperactivation of the body's immune system in the months preceding surgical intervention, a traumatic insult, is protective compared to no intervention. However, the timing of such an effect is difficult to assess; recent scholarship has demonstrated that spinal cord injury itself results in an eventual immunodeficiency mediated by autonomic dysregulation [17-18] while inflammation has also been indicated as one of the most important factors responsible for herniation regression [19].

The present study is not without limitations. The major limitation of this study is that it is retrospective. Additionally, the present study did not control for the severity of pathology or functional status before surgery; the authors hope that with cohorts greater than 20,000 each, the functional status and severity of pathology would be balanced; however, it is possible that many of those in cohort 2, those who did not receive an ESI prior to surgery, presented more acutely and thus may have bypassed the ESI prior to surgical intervention. Additionally, certain surgical interventions and spinal levels necessitate riskier approaches with longer procedures, introducing a bias towards increased complications in these patients. This study grouped several spinal surgery procedures to increase the power. However, the nuance of the pathophysiology belying the various reasons for spinal cord or vertebral disease necessitates more specific considerations. As an example, intervertebral disc degeneration is strongly associated with a pro-inflammatory state [20-22] and is likely to benefit from an ESI because degeneration results in permanent loss; on the other hand, the inflammation associated with an injury-related disc herniation often provides restorative benefits, as has been demonstrated in the spontaneous regression of certain lumbar disc herniations [19], and is thus less likely to benefit from an ESI.

## Conclusions

This study sought to interrogate the utility of epidural steroid injections in the treatment of patients undergoing spinal surgery by comparing the incidence of post-surgical mortality and morbidity in the three months after spinal surgery between patients who received an ESI in the six months prior to spinal surgery and those who did not receive an ESI in the six months prior to spinal surgery. The present results indicate that an ESI did not provide any effect on mortality, wound infection or dural injury; however, an ESI in the six months prior to spinal surgery was associated with a statistically significant decrease in the likelihood of epidural/spinal abscess in the three months after surgery.
